# Supplementary material for: Ammunition Waste Pollution and Preliminary Assessment of Risks to Child Health from Toxic Metals at the Greek Refugee Camp Mavrovouni
Source: Int J Environ Res Public Health. 2022 Aug 15;19(16):10086. doi: 10.3390/ijerph191610086 (PMC9408271; doi:10.3390/ijerph191610086)
Supplement: Supplementary file 1 [file ijerph-19-10086-s001.zip › ijerph-1811028-supplementary.pdf]

Table S1. Information on the quality of the chemical analysis. The standard reference materials (SRM) analyzed for concentration of Cu, Sb, Pb, and Bi were NCS ZC 73007 Soil, NIST SRM 2710a: Montana I Soil, Highly elevated trace element concentrations, and NIST SRM 2711a: Montana II Soil, Moderately elevated trace element concentrations. Number of replicates was four. Bias is indicated relative to certified value (*) or reference value (**); where no value is issued, (-) is displayed.

SRM	Quality indicator	Pb	Sb	Bi	Cu	U
NCS ZC 73007	Average ± SD (mg/kg)	56.3 ± 0.50	2,15 ± 0.13	1.65 ± 0.10	30.8 ± 0.50	4.98 ± 0.17
	RSD (%)	0.9	6	6.1	1.6	3.4
	Bias (%)	−7.8 *	13 **	15 *	−3.9 *	−15 *
2711a	Average ± SD (mg/kg)	1280 ± 50	22.3 ± 0.50	3.03 ± 0.22	128 ± 5.0	2.88 ± 0.13
	RSD (%)	3.9	2.2	7.3	3.9	4.4
	Bias (%)	−8.9 *	−6.5 *	-	−8.9 *	−4.3 *
2710a	Average ± SD (mg/kg)	5250 ± 100	45.5 ± 1.9	50 ± 0.0	3050 ± 58	8.40 ± 0.36
	RSD (%)	1.9	4.2	0	1.9	4.2
	Bias (%)	−4.9 *	−13 *	-	−11 *	−7.8 *

Abbreviations: NCS, NCS Testing Technology Co., Ltd., Beijing, China; NIST, National Institute of Standards & Technology, Gaithersburg, MD, U.S.A; RSD, relative standard deviation; SRM, standard reference material.

Table S2. Limit of detection (LOD) and limit of quantification (LOQ) were calculated by multiplying the standard deviations of the blank samples ($n = 6$) by three and ten, respectively. The six blank samples were taken through the whole measurement procedure, including the sample preparation steps. To express the limits on a weight/weight basis an average sample weight of 0.244 g and a dilution volume of 50 mL were used.

	Pb	Sb	Bi	Cu	U	As	Cd	Zn
**LOD (mg/kg)**	0.04	0.04	0.01	0.07	0.0002	0.02	0.002	0.9
**LOQ (mg/kg)**	0.13	0.13	0.033	0.23	0.0008	0.053	0.0071	2.9

Table S3. Average concentration of arsenic (As), cadmium (Cd), and zinc (Zn) in surface soil (0–2 cm) collected in the Greek refugee camp Mavrovouni. N indicates the number of replicates. See Figure 1 for location of sampling sites 1–8 and reference sites Ref 1 and Ref 2.

		As		Cd		Zn	
**Site #**	**N**	**Average ± SD (mg/kg)**	**RSD (%)**	**Average ± SD (mg/kg)**	**RSD (%)**	**Average ± SD (mg/kg)**	**RSD (%)**
**1**	7	12 ± 2.3	20	0.085 ± 0.0075	8.8	130 ± 10	7.7
**2**	7	15 ± 1.4	9	0.13 ± 0.0053	4.3	78 ± 2.3	2.9
**3**	7	40 ± 3.8	9.4	1.1 ± 0.21	20	100 ± 6.4	6.4
**4**	7	9.3 ± 0.48	5.2	0.097 ± 0.0024	2.5	141 ± 9.0	6.4
**5**	1	5		0.38		27	
**6**	7	13 ± 0.98	7.8	0.032 ± 0.0030	9.4	81 ± 3.3	4.1
**7**	7	18 ± 4.1	22	0.071 ± 0.026	37	87 ± 7.1	8.1
**8**	7	26 ± 3.0	12	0.080 ± 0.015	18	58 ± 2.1	3.7
**Ref 1**	7	14 ± 6.4	47	0.20 ± 0.019	9.8	59 ± 9.3	16
**Ref 2**	1	11		0.18		55	
